# Todd’s Paralysis Simulating Acute Ischemic Stroke: A Case Report

**DOI:** 10.7759/cureus.110685

**Published:** 2026-06-11

**Authors:** Poleth G Medrano-Madrid, Edgar A Espinoza-Beltrán, Jose M Espinoza-Beltrán, Erika M Espinoza-Beltrán

**Affiliations:** 1 Internal Medicine, Servicios de Salud del Instituto Mexicano del Seguro Social para el Bienestar, Hospital General de Culiacán, Culiacán, MEX; 2 Radiology, Centro de Investigación y Docencia en Ciencias de la Salud, Hospital Civil de Culiacán, Culiacán, MEX; 3 Neurology, Faculty of Medicine, Universidad Autónoma de Sinaloa, Culiacán, MEX

**Keywords:** acute ischemic stroke, epilepsy, postictal aphasia, seizure disorder, stroke mimic, todd's paralysis

## Abstract

Todd’s paralysis is a transient focal neurological deficit following epileptic seizures that may closely mimic an acute ischemic stroke, posing a diagnostic challenge in the emergency department. We report the case of a 32-year-old woman with a history of epilepsy who presented with persistent expressive aphasia and right hemiparesis after experiencing multiple generalized tonic-clonic seizures. On admission, her National Institutes of Health Stroke Scale (NIHSS) score was 6, initially raising strong suspicion for an acute ischemic cerebrovascular event. Noncontrast cranial computed tomography (CT) showed no intracranial hemorrhage or early ischemic changes. Due to the persistence of focal deficits, brain magnetic resonance imaging (MRI) was performed, which demonstrated no evidence of acute cerebral infarction or structural abnormalities. Electroencephalography (EEG) demonstrated focal epileptiform activity with secondary generalization. Following optimization of her antiseizure regimen with the addition of valproate, the patient achieved complete seizure control and full resolution of all neurological deficits, establishing the diagnosis of Todd’s paralysis with postictal aphasia. This case underscores the importance of considering Todd’s paralysis in the differential diagnosis of acute focal neurological deficits, particularly in patients with a history of epilepsy and prolonged postictal symptoms. In resource-limited settings where advanced perfusion imaging may be unavailable, integrating clinical findings, conventional neuroimaging, and electroencephalographic evaluation can provide sufficient diagnostic confidence to distinguish severe postictal deficits from acute ischemic stroke and avoid unnecessary or potentially hazardous interventions.

## Introduction

Todd’s paralysis is a postictal phenomenon characterized by transient focal neurological deficits following epileptic seizures. It commonly presents as hemiparesis, aphasia, or focal sensory disturbances, which may closely mimic acute ischemic stroke and create significant diagnostic dilemmas in acute care settings [1]. Distinguishing stroke from stroke mimics at the bedside remains difficult, prompting the development of clinical assessment tools and scoring systems to improve diagnostic accuracy in emergency settings [2]. Stroke mimics represent a substantial proportion of emergency stroke activations, with epileptic seizures and postictal states identified among the most frequent etiologies [3,4].

When focal neurological deficits persist after a seizure, differentiating Todd’s paralysis from a true cerebrovascular event becomes challenging, yet it carries important diagnostic and therapeutic implications. While initial non-contrast computed tomography (CT) findings are frequently normal in both conditions, advanced diagnostic modalities such as brain magnetic resonance imaging (MRI) and electroencephalography (EEG) play important roles in diagnostic differentiation [1,5].

Here, we present the case of a patient with known epilepsy who developed prolonged expressive aphasia and right-sided hemiparesis following recurrent seizures, initially raising strong suspicion for acute ischemic stroke. While Todd’s paralysis is conceptually well recognized, its occurrence as a profound stroke mimic within acute care workflows poses a critical double-edged dilemma: the risk of administering potentially hazardous reperfusion therapies to a postictal patient versus the catastrophic delay of thrombolysis in a true cerebrovascular event. This challenge is significantly amplified in resource-limited settings where advanced perfusion imaging is unavailable. By examining the chronological clinical reasoning, conventional multimodal neuroimaging findings, and electroencephalographic integration used to navigate this diagnostic ambiguity, this report aims to provide practical educational insights into distinguishing severe postictal deficits from hyperacute ischemic stroke at the bedside.

## Case presentation

A 32-year-old woman with class III obesity, weighing 80 kg, and a history of epilepsy diagnosed at five years of age presented to the emergency department. Her home maintenance regimen consisted of phenytoin 100 mg every eight hours and levetiracetam 1500 mg orally every 12 hours. She denied any history of cardiovascular disease, hypertension, diabetes mellitus, allergies, prior blood transfusions, or substance use. Her surgical history was notable only for an uncomplicated cesarean section at 17 years of age. Medication adherence and serum antiseizure drug levels could not be confirmed upon admission; however, subsequent review of the clinical history strongly suggested poor treatment adherence as the most likely precipitating factor for the breakthrough seizures.

Approximately eight hours prior to evaluation, the patient experienced a sudden onset of generalized tonic-clonic seizures without prodromal symptoms. According to family members, the initial episode involved an abrupt loss of consciousness, ocular deviation, and cyanosis, followed by generalized tonic and clonic movements lasting approximately five minutes and resolving spontaneously. Minutes later, she experienced a second seizure with identical clinical characteristics. Subsequently, she manifested a postictal state lasting roughly 20 minutes, characterized by stupor and disorientation. Upon regaining consciousness, her family noted persistent expressive aphasia and right-sided weakness, prompting transfer to our center.

On initial emergency evaluation, her vital signs were within normal limits. The neurological examination revealed prominent expressive aphasia, dysarthria, right central facial palsy, and right hemiparesis, with muscle strength graded as 3/5 on the Medical Research Council (MRC) scale [6]. Hypoesthesia was also noted on the right side of her body. Her baseline National Institutes of Health Stroke Scale (NIHSS) score was 6 [7]. Notably, the patient was evaluated approximately eight hours after symptom onset, placing her well outside the standard 4.5-hour therapeutic window for intravenous thrombolysis.

Given the acute focal deficits and the high suspicion of acute ischemic stroke, an urgent non-contrast cranial CT scan was performed, demonstrating no evidence of intracranial hemorrhage or early signs of acute ischemia (Figure 1).

**Figure 1:**
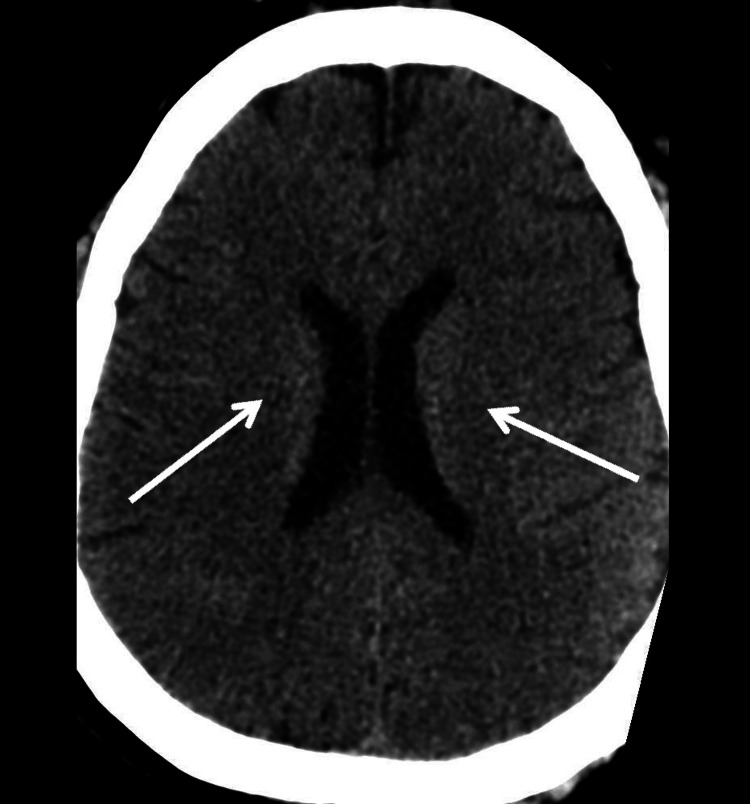
Non-contrast cranial CT White arrows highlight representative cerebral regions with preserved gray-white matter differentiation and no evidence of intracranial hemorrhage or early ischemic changes. CT, computed tomography

Baseline laboratory investigations obtained upon hospital admission included a complete blood count, serum chemistry, electrolytes, total cholesterol, triglycerides, and renal function tests, all of which were largely within normal reference ranges (Table 1). These investigations were systematically prioritized to exclude acute, potentially reversible systemic derangements, such as profound hypoglycemia, severe hyponatremia, or uremic encephalopathy, that could independently lower the seizure threshold or mimic persistent focal neurological deficits of vascular origin.

**Table 1:** Laboratory findings at admission and follow-up evaluation

Parameter	Admission	Follow-up	Reference range
Glucose (mg/dL)	98.59	88.60	70 - 105
Urea (mg/dL)	10.69	11.71	10 - 55
Creatinine (mg/dL)	0.59	0.60	0.60 - 1.10
Blood urea nitrogen (mg/dL)	5.00	5.47	7 - 18
Sodium (mmol/L)	144.00	142.86	135 - 148
Potassium (mmol/L)	4.16	4.38	3.5 - 5.3
Chloride (mmol/L)	106.04	96.95	95 - 110
Phosphorus (mg/dL)	3.19	3.22	2.5 - 4.9
Magnesium (mg/dL)	1.82	1.85	1.8 - 2.4
Calcium (mg/dL)	8.55	8.95	8.5 - 10.1
Total cholesterol (mg/dL)	115.12	114.53	140 - 200
Triglycerides (mg/dL)	163.17	-	35 - 150
Hemoglobin (g/dL)	12.1	12.9	12 - 18
Hematocrit (%)	37.5	39.4	37 - 51
Leukocytes (×10³/µL)	9.78	9.9	5 - 10
Segmented neutrophils (%)	55.0%	70%	37 - 95
Lymphocytes (%)	35.8%	21.0%	20 - 45
Platelets (×10³/µL)	324	361	150 - 450

Initial emergency antiseizure management involved intravenous diazepam 10 mg as rescue therapy, followed by an intravenous loading dose of phenytoin calculated at 15 mg/kg (1200 mg). Maintenance therapy was continued with phenytoin 100 mg intravenously every eight hours alongside her baseline regimen of levetiracetam 1500 mg orally every 12 hours. Despite dual therapy, the patient experienced recurrent breakthrough seizures during the first 24 hours of hospitalization. This prompted the addition of oral valproate 600 mg every 12 hours, which successfully achieved complete seizure control.

Due to the persistence of her focal neurological deficits and to definitively rule out a cerebrovascular event, a brain MRI with and without contrast was obtained on the second hospital day. Fluid-attenuated inversion recovery (FLAIR), diffusion-weighted imaging (DWI), and apparent diffusion coefficient (ADC) sequences revealed no evidence of acute cerebral infarction, structural lesions, or intracranial masses (Figure 2).

**Figure 2:**
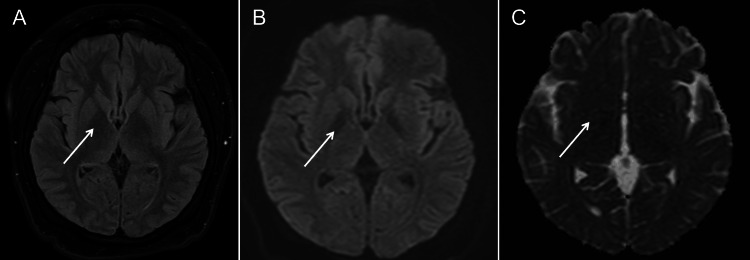
Brain MRI performed on the second hospital day (A) FLAIR sequence showing no focal cortical edema or structural abnormalities. (B) DWI sequence demonstrating no areas of restricted diffusion suggestive of acute ischemia. (C) ADC map confirming the absence of acute cerebral infarction. White arrows highlight representative regions without abnormal findings across all sequences. MRI, magnetic resonance imaging; FLAIR, fluid-attenuated inversion recovery; DWI, diffusion-weighted imaging; ADC, apparent diffusion coefficient

Follow-up laboratory studies obtained on the same day showed stable renal function, normal electrolyte levels, and no significant metabolic abnormalities that could otherwise account for her persistent neurological deficits (Table 1). A concurrent EEG revealed focal epileptiform discharges associated with regional slowing, findings consistent with an epileptogenic focus and focal seizure activity with secondary generalization.

By the third hospital day, the patient exhibited complete and spontaneous neurological recovery. Her expressive aphasia and dysarthria resolved entirely, facial asymmetry disappeared, and full motor strength (5/5) was restored in her right extremities. Based on the transient nature of the deficits, complete clinical resolution, negative multimodal neuroimaging, and supportive electroencephalographic data, a final diagnosis of Todd’s paralysis with postictal aphasia secondary to recurrent epileptic seizures was established. The patient was safely discharged home on an optimized oral regimen of phenytoin, levetiracetam, and valproate. At her initial outpatient follow-up with the Neurology Department, she remained entirely seizure-free and neurologically intact.

## Discussion

Todd’s paralysis must remain an important differential diagnosis when managing patients who exhibit acute focal neurological deficits following an epileptic event. Because its clinical presentation, severity, and duration vary widely, it represents an important stroke mimic, complicating early decision-making in emergency departments.

In this patient, the combination of expressive aphasia and right-sided motor deficits strongly raised suspicion for an acute ischemic event. The presence of expressive aphasia was particularly concerning, as it suggested involvement of the dominant cerebral hemisphere. Distinguishing stroke from stroke mimics at the bedside remains challenging, prompting the development of clinical scoring systems, telemedicine-based assessment tools, and emergency stroke screening scores to improve diagnostic accuracy [2]. Classic focal signs such as hemiparesis and speech disturbances are commonly observed in both confirmed ischemic strokes and stroke mimics, significantly limiting the diagnostic accuracy of clinical examination alone [3].

In our case, the persistent nature of the neurological deficits despite a normal initial non-contrast CT scan necessitated further neuroimaging evaluation. Differentiating a true cerebrovascular event from a stroke mimic at the bedside continues to represent a major clinical challenge, and administering thrombolytics under diagnostic uncertainty without definitive neuroimaging criteria may expose patients to unnecessary hemorrhagic risks [8]. Recent studies have further explored the frequency and pathophysiology of Todd’s paralysis, emphasizing its variable clinical manifestations and postictal mechanisms [9]. Moreover, advanced imaging studies have described distinct patterns of transient focal hypoperfusion or hyperperfusion during perfusion imaging in Todd’s paralysis, reflecting dynamic postictal cerebrovascular shifts that may help distinguish these cases from ischemic penumbra [10]. However, advanced perfusion imaging was not available at our institution during the emergency phase.

This limitation highlights an important practical lesson for clinicians working in resource-constrained settings. Although brain MRI, including DWI and ADC sequences, demonstrated no evidence of restricted diffusion or acute cerebral infarction, the absence of perfusion imaging required greater reliance on a multimodal diagnostic approach. In our patient, the EEG revealed focal epileptiform discharges associated with regional slowing, findings consistent with an epileptogenic focus and focal seizure activity with secondary generalization. In the setting of transient aphasia and hemiparesis, the absence of acute ischemic lesions on neuroimaging and complete neurological recovery, these neurophysiological findings supported a postictal etiology and were concordant with the clinical evolution characteristic of Todd’s paralysis. Together with the negative conventional neuroimaging findings, these observations strongly supported a postictal rather than vascular mechanism. Furthermore, recurrent breakthrough seizures despite maintenance therapy with phenytoin and levetiracetam justified escalation of antiseizure treatment with valproate, after which complete seizure control and neurological recovery were achieved. Given that the patient presented well outside the therapeutic time window, intravenous thrombolysis was not indicated. Under these circumstances, the absence of perfusion imaging reinforced the need for close clinical observation and optimized antiseizure management while alternative diagnoses were systematically evaluated.

Early and accurate recognition of Todd’s paralysis carries major therapeutic implications. Previous reports have demonstrated that a considerable proportion of patients with stroke mimics may inadvertently receive intravenous thrombolysis because of the urgency of the initial diagnosis [8]. While thrombolytic administration in non-stroke patients appears to have a relatively low complication rate, it nonetheless exposes patients to avoidable hemorrhagic risks and delays appropriate management. Fortunately, our patient was outside the thrombolysis window, avoiding this risk, but her presentation still required urgent diagnostic clarification.

Furthermore, atypical and prolonged presentations of Todd’s paralysis continue to be reported in the literature [11], reinforcing the need to maintain a high index of suspicion when patients with known epilepsy develop persistent focal neurological deficits. In such scenarios, careful integration of clinical history, conventional neuroimaging findings, electroencephalographic data, and short-term neurological evolution may provide sufficient diagnostic confidence even when advanced perfusion studies are unavailable. Our case illustrates how this multimodal approach can facilitate appropriate antiseizure management while avoiding unnecessary interventions.

## Conclusions

Todd’s paralysis remains a challenging differential diagnosis in emergency settings when persistent postictal aphasia and hemiparesis closely mimic acute ischemic stroke. While advanced perfusion modalities represent the preferred diagnostic approach in diagnostically ambiguous cases, this report illustrates that a clinically reliable diagnosis may be achieved in resource-limited environments without exposing patients to potentially unnecessary or hazardous interventions. This can be accomplished through the systematic integration of a detailed epilepsy history, exclusion of acute ischemia using conventional sequential neuroimaging (including DWI/ADC sequences), and electroencephalographic evaluation. Ultimately, a normal initial cranial CT scan coupled with prolonged focal neurological deficits in a patient with suspected antiseizure medication nonadherence should prompt strong consideration of Todd’s paralysis, with close clinical follow-up serving to document complete spontaneous neurological recovery.
